# Additive Manufacturing of Personalized Pharmaceutical Dosage Forms via Stereolithography

**DOI:** 10.3390/pharmaceutics11120645

**Published:** 2019-12-03

**Authors:** Andrew V. Healy, Evert Fuenmayor, Patrick Doran, Luke M. Geever, Clement L. Higginbotham, John G. Lyons

**Affiliations:** 1 Materials Research Institute, Athlone Institute of Technology, Dublin Road, Athlone, Co., Westmeath N37 HD68, Ireland; andrewhealy@research.ait.ie (A.V.H.); e.fuenmayor@research.ait.ie (E.F.); lgeever@ait.ie (L.M.G.); chigginbotham@ait.ie (C.L.H.); 2 Applied Polymer Technologies Gateway, Athlone Institute of Technology, Dublin Road, Athlone, Co., Westmeath N37 HD68, Ireland; patrickdoran@ait.ie; 3 Faculty of Engineering and Informatics, Athlone Institute of Technology, Dublin Road, Athlone, Co., Westmeath N37 HD68, Ireland

**Keywords:** stereolithography, three-dimensional printing, additive manufacturing, personalized medicine, 3D printed oral dosage forms, drug delivery, sustained drug release tablets, photopolymerization, paracetamol (acetaminophen), aspirin (acetylsalicylic acid)

## Abstract

The introduction of three-dimensional printing (3DP) has created exciting possibilities for the fabrication of dosage forms, paving the way for personalized medicine. In this study, oral dosage forms of two drug concentrations, namely 2.50% and 5.00%, were fabricated via stereolithography (SLA) using a novel photopolymerizable resin formulation based on a monomer mixture that, to date, has not been reported in the literature, with paracetamol and aspirin selected as model drugs. In order to produce the dosage forms, the ratio of poly(ethylene glycol) diacrylate (PEGDA) to poly(caprolactone) triol was varied with diphenyl(2,4,6-trimethylbenzoyl)phosphine oxide (Irgacure TPO) utilized as the photoinitiator. The fabrication of 28 dosages in one print process was possible and the printed dosage forms were characterized for their drug release properties. It was established that both drugs displayed a sustained release over a 24-h period. The physical properties were also investigated, illustrating that SLA affords accurate printing of dosages with some statistically significant differences observed from the targeted dimensional range, indicating an area for future process improvement. The work presented in this paper demonstrates that SLA has the ability to produce small, individualized batches which may be tailored to meet patients’ specific needs or provide for the localized production of pharmaceutical dosage forms.

## 1. Introduction

Three-dimensional printing (3DP) is an additive manufacturing (AM) process which involves the fabrication of an object or structure from a digital form by the deposition or binding of materials layer-by-layer [1,2,3,4]. Over the last few decades, 3DP has received extraordinary attention, particularly for its use within both the biomedical and pharmaceutical sectors. The introduction of 3DP has led to a potential shift in the way in which drug dosage forms may be manufactured. Further, with an ever-ageing population and the growing understanding of pharmacogenomics, there is an increased requirement for personalized medicine to be optimized. 3DP can facilitate the fabrication of dosage forms of complex geometries [5,6,7] with immediate [8,9,10,11,12] or modified [8,9,11,12] drug release profiles, some of which may contain multiple drugs [9,13,14,15]. These characteristics allow greater flexibility in the manufacturing of dosage forms, which would not otherwise be possible utilizing conventional manufacturing technologies [16,17], thus permitting the change from the current “one size fits all” approach [18]. This may revolutionize the production of personalized medicines [19,20] which will be of particular benefit in the treatment of patients who are known to have a pharmacogenetic polymorphism or are currently being treated with drugs which have narrow therapeutic indices [21].

Currently, there are numerous AM technologies available on the market, such as stereolithography (SLA), fused filament fabrication (FFF), and selective laser sintering (SLS) among others, many of which have been explored as potential fabrication approaches for drug delivery. For example, SLA has been investigated by Martinez and researchers [22] who examined the ability SLA offers in the fabrication of controlled release dosage forms and found that by modifying the water content in the formulation it was possible to alter the rate at which the drug was released. In a follow up study, Martinez et al. [6] explored the effect of geometry on drug release from dosage forms with their results indicating that out of all the geometric parameters examined the surface area to volume ratio had the greatest impact regarding drug release kinetics. Meanwhile, Fuenmayor and co-workers [23] evaluated the considerations that material choice plays in the FFF of dosage forms. Further to this, they compared FFF to direct compression (DC) and found that the manufacturing method utilized determined the drug release properties with FFF dosage forms taking considerably longer to release the active drug than that of DC, demonstrating the potential FFF has to offer for extended/modified release applications. In addition to this, Fuenmayor et al. [12] further compared FFF to two alternative manufacturing techniques, DC and injection molding, to conclude that through the manipulation of the printing parameters it was possible to alter the drug release characteristics, which would not be possible in the other two techniques they explored. Furthermore, Fina et al. [8] conducted a study to evaluate if the alternative AM technique SLS could potentially be exploited in the fabrication of oral dosage forms using pharmaceutical grade excipients. From their study, the authors [8] deduced that SLS has the capacity to yield personalized dosage forms and with careful selection of excipients it is possible to fabricate dosage forms of either immediate-release or modified-release. In addition to this study, Fina and researchers [10] built on their knowledge of the SLS process by fabricating orally disintegrating dosage forms that had increased drug release properties which were dictated by an adjustment of the printing parameters with accelerated release observed in a faster laser scanning speed, illustrating that the manipulation of printing parameters is not just limited to the FFF technique. All of the aforementioned studies and AM techniques employed highlight the current possibilities available to be exploited in both the area of drug delivery and personalized medicine.

However, the most remarkable use of 3DP in drug delivery to date is the first U.S. Food and Drug Administration (FDA) approved 3D printed medicine, Spritam^®^ (levetiracetam), which is a rapidly disintegrating oral medicine [3,24]. The innovative technology employees a powder-based method of 3DP allowing dosage forms to be fabricated in various strengths, namely 250, 500, 750, and 1000 mg, [25] illustrating the potential benefits that it offers in terms of drug delivery with the possibility of manufacturing bespoke drug formulations which may be dependent on the patient’s specific needs, further proving that it may offer the flexibility to prepare small batch sizes of personalized dosage forms [15,26,27].

The purpose of this study was to evaluate the possibility of fabricating drug dosage forms utilizing the AM technique of stereolithography. The stereolithography process was successful in the fabrication of the tablets with the tablets being characterized by a variety of testing procedures. Fourier transform infrared (FTIR) spectroscopic analysis was used to determine the chemical structure of the printed dosages and also evaluate the extent of the reaction, while thermal characterization assessed if the active drug was dissolved within the photopolymer solution. Furthermore, the tablets were analyzed for their dimensional accuracy, weight variation, and drug release kinetics with paracetamol and aspirin being selected as the model drugs for the study. Scanning electron microscopy (SEM) was also utilized to investigate the structure of the tablets.

## 2. Materials and Methods

### 2.1. Materials

Poly(ethylene glycol) diacrylate (PEGDA, average M_w_ 700, Batch number: MKBW9043V), paracetamol (acetaminophen, Batch number: MKCD6375), aspirin (acetylsalicylic acid, Batch number: SLBV2290), methanol and 2-propanol were purchased from Sigma Aldrich, Wicklow, Ireland). Poly(caprolactone) Triol, (PCL Triol), Capa™ 3031 (average M_w_ 300, Lot number: WBY 000428) was received as a gift from Perstorp UK Ltd., Cheshire, UK. Diphenyl(2,4,6-trimethylbenzoyl)phosphine oxide (Irgacure TPO, Batch number: 50019766) was also received as a gift from BASF, (Ludwigshafen, Germany). All salts required in the preparation of the dissolution media were also procured from Sigma Aldrich (Wicklow, Ireland). All materials were used as received unless otherwise stated.

### 2.2. Stereolithography

#### 2.2.1. Preparation of Photopolymer Formulations

The photopolymer formulations were prepared as outlined in Table 1. The PEGDA and PCL Triol were added together and mixed for a duration of 2 h, after which the photoinitiator, Irgacure TPO, was added at a concentration of 1.00% (*w/w*). The solution was mixed thoroughly for a duration of 8 h to ensure that the photoinitiator had completely dissolved. Paracetamol or aspirin was then added to the solution at a concentration of 2.50% (*w/w*) or 5.00% (*w/w*) as shown in Table 1 with the solution constantly mixed until complete dissolution of the drug. All mixing of solutions was carried out using a magnetic stirrer and mixed at room temperature. In addition to this, all formulations were protected from light at all stages of production, mixing, and storage by the use of amber Duran bottles. Prior to printing, the prepared respective photopolymer solution was loaded into the printing tray.

#### 2.2.2. Stereolithographic Printing

**Figure 1:**
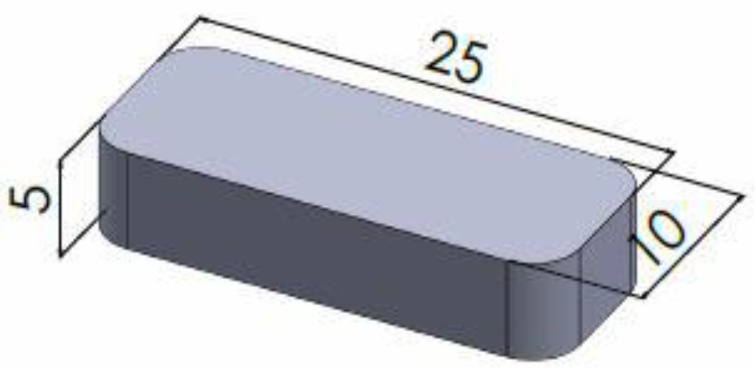
Computer-aided design (CAD) of the printed oral dosage form (length 25.00 mm, width 10.00 mm, height 5.00 mm).

All stereolithographic printing was conducted by utilization of a Formlabs Form 2 SLA 3D printer (Formlabs Inc., Somerville, MA, USA) which is equipped with 405 nm wavelength light source. The SLA printer has the capabilities of fabricating objects with a resolution that allows for a layer thickness of 25, 50, or 100 µm. The design for the 3D printed oral dosage form Figure 1 was designed in SolidWorks 2017 (Dassault Systèmes, Waltham, MA, USA) and exported as a stereolithography file (STL) to the 3D printer software (PreForm Software version 2.15.1). In order to print using the formulated photopolymer resin, “Open Mode” was enabled on the printer, which deactivates the dispensing of resin and the resin heating and wiping capabilities.

### 2.3. Spectroscopic Analysis of Photoinitiator

#### 2.3.1. UV/Vis Spectrophotometry

The UV/Vis spectra of the photoinitiator, Irgacure TPO was measured on a UV/Vis spectrophotometer (Shimadzu UV-1280, Milton Keynes, UK). The spectra were recorded for three different concentrations of the photoinitiator, 0.10%, 0.50%, and 1.00%, in two different solvents, methanol and 2-propanol, by conducting a wavelength scan ranging from 450–325 nm with the spectrophotometer being blanked with the respective solvent prior to the analysis being conducted.

#### 2.3.2. Fourier Transform Infrared Spectrometry

Fourier transform infrared (FTIR) spectroscopy was carried out by the utilization of a Spectrum One FTIR spectrometer (Perkin Elmer, Dublin, Ireland) which incorporates a universal Attenuated Total Reflectance (ATR) sampling accessory. All data was recorded at ambient temperature, in the spectral range 4000–650 cm^−1^, utilizing 16 scans per cycle at 4 cm^−1^ resolution and a fixed compression force of 80 N. Subsequent analysis was carried out on the Spectrum software.

### 2.4. Thermal Characterization

The printed dosage forms were characterized by the utilization of differential scanning calorimetry (DSC), DSC 2920 Differential Scanning Calorimeter (TA Instruments). Samples of between 6.0–8.0 mg were weighed out using a Sartorius analytical balance with a resolution of 1 × 10 ^−5^ g and placed in non-hermetic aluminum pans, which were crimped prior to testing with an empty crimped aluminum pan being used as the reference pan. Calorimetry scans were carried out by heating each sample at a rate of 10 °C/min from −20 °C to 200 °C.

However, for the thermal characterization of paracetamol, the second heating cycle was also evaluated in order to ascertain what polymorph the paracetamol existed in.

All DSC scans were carried out under a 30 mL/min flow of nitrogen. Prior to conducting the characterization, the DSC was calibrated using indium as standard.

### 2.5. Tablet Characterization

In order to evaluate the dimensional accuracy that the SLA printing process affords printed dosage forms (*n* = 10) were measured for their length, width and height using digital calipers with Figure 1 showing the anticipated measurements that should be obtained from the printed dosage form. A two-way ANOVA was used to analyze the variance between tablet dimensions with the Dunnett’s multiple comparisons test used determine the significant difference between expected dimensions and printed dosage forms with differences being considered significant when *p* ≤ 0.05. Furthermore, the printed dosage forms were analyzed for their uniformity of weight to evaluate the consistency of the printing process with ten tablets taken and measured on a Sartorius analytical balance with a resolution of 1 × 10 ^−5^ g.

### 2.6. Dissolution Analysis

Drug dissolution profiles for the printed dosage forms were obtained by utilization of a Distek dissolution system 2100B with a Distek temperature control system TCS 0200B (Distek Inc., North Brunswick, NJ, USA) according to USP dissolution apparatus I. The dosage forms were investigated in 0.2 M hydrochloric acid, pH 1.2 with 900 mL of media used per vessel. All testing was conducted at 37 ± 0.5 °C with the stir rate being 50 RPM. At pre-determined time intervals, 5 mL was withdrawn from each vessel and replaced with pre-heated dissolution media. The withdrawn samples were filtered through 0.45 µm filters with drug release being determined at 243.2 nm for paracetamol and 233.2 nm for aspirin by performing UV spectroscopy on a Shimadzu UV-1280 UV-VIS spectrophotometer (Shimadzu, Milton Keynes, UK). Drug dissolution studies were conducted in sextuplicate with the average percentage of drug release as a function of time being determined. A two-way ANOVA with multiple comparisons among subgroups was performed using a Bonferroni post-hoc test to differentiate drug release curves with differences being considered significant when *p* ≤ 0.05.

### 2.7. Scanning Electron Microscopy

Surface and cross-section images at a range of magnifications of the printed dosage forms were taken by with a Mira SEM (Tescan, Oxford Instruments, Cambridge, UK). The samples were placed on aluminum stubs using double-sided conductive carbon tape which were then sputter-coated with gold using an SCD 005 sputter coater (Bal-Tec, Balzers, Liechtenstein) for 110 s at 0.1 mBar vacuum prior to observation. All images were generated using an accelerating voltage of 20 kV and were recorded at magnifications of 300, 500 and 1000×.

### 2.8. Statistical Analysis

Data handling and analysis were performed using GraphPad Prism (GraphPad Prism version 8.0.1 for Windows, GraphPad Software, San Diego, CA, USA). All test data was inputted into the software with the mean and standard deviations calculated for the replicate data sets. All data were assessed for normality of distribution after which the relevant statistical analysis was performed with differences being considered significant when *p* ≤ 0.05. All data in figures are presented as mean with error bars representing standard deviation unless otherwise stated.

## 3. Results and Discussion

### 3.1. Stereolithographic Printing Process

**Figure 2:**
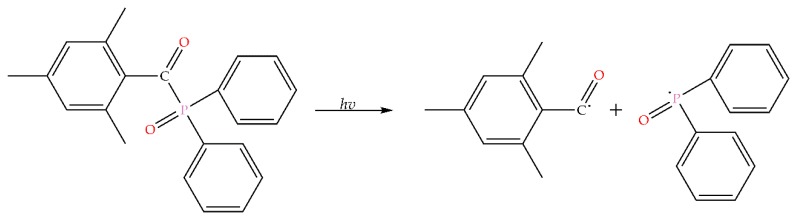
Norrish Type 1 photocleavage of Irgacure TPO.

The dosage forms in this study were fabricated utilizing the AM technique of SLA. SLA is a method in which objects may be fabricated or produced by using focused ultraviolet (UV) light and photocurable materials to produce solid 3D forms due to a photopolymerization reaction occurring. Photopolymerization is the use of light in order to initiate a polymerization reaction, thus resulting in the conversion of a liquid monomer to a solid polymer through a chemical reaction taking place [28]. The polymerization reaction is initiated by the addition of light-sensitive compounds called photoinitiators, which become active under the appropriate wavelengths, creating free radicals due to the conversion of the absorbed light energy [29]. These free radicals are then consumed in the reaction converting the liquid monomer into a solid-state, which may be referred to as a crosslinked hydrogel [28,29]. The photoinitiator used within this study is Irgacure TPO, a Norrish Type 1, photoinitiator which is a single-molecule and undergoes photocleavage into radical fragments when exposed to light from an appropriate wavelength as illustrated in Figure 2 [30,31].

In order to fabricate the drug dosage forms, it was necessary to prepare the photopolymer solutions to be loaded into the SLA 3D printer which was carried out by altering the ratio of PCL Triol to PEGDA in the formulation with the ratios being chosen based on previous experimentation work in our lab and on the rheological profiling of existing formulations.

PCL Triol is a biodegradable, semi-crystalline, aliphatic polyester, with low molecular weight and melting point which has been investigated to a great extent as a biomaterial as well as in drug delivery applications [32,33,34]. One reason for this is the degradation products of PCL being naturally occurring metabolites in the human body with sutures composed of PCL being approved by the US Food and Drug Administration (FDA) [35]. Further to this, low molecular weight PCL Triols have been investigated for their potential to act as plasticizers [33,36,37] with Kanis et al. [34] indicating that it is the three hydroxyl groups present which allow it to act as a plasticizer. Furthermore, PCL Triols due to their nature do not possess photopolymerizable terminal groups and therefore cannot crosslink on their own which is why PCL derivatives have been developed [38,39,40]. Like PCL Triol, poly(ethylene glycol) (PEG) itself cannot crosslink [41] and has also been investigated for use as a plasticizer [42,43] and in drug delivery applications [11,44,45,46]. Martinez et al. [22] conducted a study in which they added PEG 300 to the resin formulation and due to its inability to crosslink it would allow for a reduction in the intermolecular forces between the polymer chains, resulting in greater drug release.

Poly(ethylene glycol) (PEG) is a well-established synthetic polymeric material with PEG based hydrogels being well documented within the literature [29,47,48,49,50] owing to the exceptional properties they afford, such as being nontoxic, biocompatible, and having notable tunability, in addition to being approved by the FDA for use in a variety clinical uses [51,52]. PEGDA has been shown to be a beneficial polymer in drug delivery applications and was investigated by McAvoy et al. [53] to evaluate its potential to fabricate implants via UV polymerization in the release of both small and large drug molecules with the former being released at a greater rate than that of the latter. This indicates that by the careful selection of drugs it may be feasible to manipulate and control the rate at which they are released from the fabricated dosage form.

**Figure 3:**
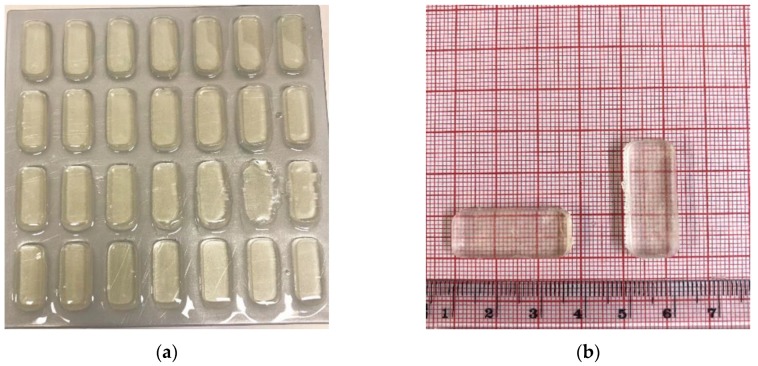
(**a**) Complete batch of printed dosage forms fabricated via SLA 3D printing (*n* = 28) (**b**) single dosage form.

The model drugs, paracetamol and aspirin, were found to easily dissolve in their respective photopolymer solutions with the colors of both solutions being clear and the 3DP dosage forms displaying the same color as in Figure 3a,b. Although the concentrations of the model drugs were low the authors selected these concentrations based on previously published contributions with Goyanes et al. [20] fabricating topical drug delivery systems with the incorporation of 2.00% *w/w* salicylic acid the primary metabolite of aspirin. In addition to this, Wang et al. [41] and Martinez et al. [6] both fabricated dosage forms with the loading of paracetamol being 5.90% *w/w* and 4.00% *w/w* respectively. While the concentrations utilized do not have immediate clinical relevance, the formulations are intended to demonstrate the capability of the AM technique for bespoke batch production within a clinical setting. Despite this, the authors do believe that drug concentration could be increased, with Martinez et al. [54] incorporating 10.00% *w/w* of both model drugs used in this study, in addition to four other model drugs, to fabricate a multi-layered polypill via SLA. However, the effect on the increased drug concentration and thus decreased polymer concentration would need to be tested in order to identify how it would affect the printing process, curing process and drug release mechanism. Paracetamol and aspirin were identified as model drugs for this work, in part because of the difference in pKa values, 9.5 and 3.5 respectively [55,56], of the two active ingredients. A rectangular shape was selected as the geometry to be printed, as shown in Figure 3. However, 3DP has the capabilities of fabricating dosage forms of varied and complex shapes which has been demonstrated by Martinez et al. [6] who evaluated the role that geometry plays in drug release from printed dosage forms.

As can be observed in Figure 3a, it was possible to fabricate 28 tablets in one single print illustrating the potential the 3DP affords in the area of personalized medicine specifically if the patient requires one dosage per day meaning that it is possible to fabricate a month’s supply in one print. This finding has the possibility to revolutionize how pharmaceuticals are fabricated, and in particular when developing drug delivery methods for the treatment of rare diseases, as there is little to no waste from the printing process. Furthermore, through the manipulation of the dimensional design parameters of the dosage form, it may also be feasible to fabricate an amount greater than what was achieved in this study, 28 dosage forms, which would be advantageous if the patient was required to take more than one dose per day.

### 3.2. Spectroscopic Analysis of Photoinitiator

**Figure 4:**
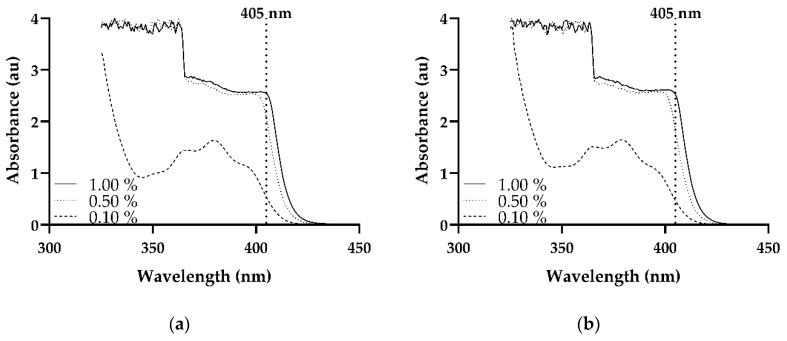
Absorbance spectra of photoinitiator at varying concentrations in (**a**) 2-propanol and (**b**) methanol.

This study utilized Irgacure TPO which is a highly efficient photoinitiator that is used in the initiation of free radical polymerization [57] with McDermott et al. [58] reporting that it absorbs radiation in the region of 420–340 nm, making it ideal for use in the SLA process as the wavelength utilized by the SLA printer is 405 nm, as reported by Dizon et al. [59]. The UV/Vis spectra of the photoinitiator Irgacure TPO was measured at three different concentrations, namely 0.10%, 0.50%, and 1.00%, and in two different solvents, 2-propanol and methanol, to establish if the photoinitiator has the capabilities of absorbing in the operating range of the SLA printer. As can be observed in the spectra, as shown in Figure 4a,b, all three concentrations in both solvents absorb in the desired range, 405 nm. In both solvents, the TPO at a concentration of 0.10% showed a rather low absorption at 405 nm when compared to the other two concentrations, 0.50% and 1.00%. Furthermore, Arikawa et al. [60] evaluated a number of photoinitiators and light curing units and concluded that both TPO and the violet-LED light was the most optimum in the polymerization of a mixture of bisphenol A-glycidyl methacrylate (Bis-GMA) and triethylene glycol dimethacrylate (TEGDMA).

Despite only a slight variance being apparent in the absorbance value between the concentrations, 0.50% and 1.00%, the latter concentration was selected for the study conducted. The rationale for selecting this concentration, 1.00%, is owing to the fact that if there is insufficient photoinitiator incorporated in the formulation this may result in surface cure problems. However, on the other hand, if too high a concentration is selected, high levels of surface crosslinking may occur which will prevent the UV light used in the printing process from effectively penetrating to the lower layers which may result in inadequate curing. Although the loading of 1.00% may seem high there are a number of publications in the literature utilizing higher concentrations. In a publication by Steyrer and researchers [61], they illustrated the possibility of using a concentration loading of 1.18 wt% of Omnirad TPO-L (Ethyl (2,4,6-trimethylbenzoyl) phenylphosphinate) in the fabrication of 3D printed part on a 405 nm Digital Light Processing (DLP) printer, and although it may not be the same process used in this study it does, however, rely on a similar technology using UV light to cure the photopolymer. In addition to this, a concentration in excess of what is proposed in this study was utilized by Asikainen et al. [62] in which they assessed lidocaine drug release from photo-crosslinkable PCL scaffolds fabricated by SLA technology.

However, the photoinitiator used in the studies by Steyrer and co-workers [61] and Asikainen and researchers [62] was an acylphosphine oxide derivative similar to that of Irgacure TPO used in this study. Furthermore, Bausch & Lomb Incorporated. filed a patent in 2010 utilizing a 1.50 wt.% of Lucirin TPO, now called Irgacure TPO, in the formulation of contact lenses [63]. In addition to this, Martinez et al. [6] also carried out a study, to explore the role that geometry plays in the release of drugs from printed tablets, using the same photoinitiator that is to be used within this study at a concentration of 2.00% *w/w*. Additionally, both the photoinitiator, Irgacure TPO, and SLA printer, Formlabs Form 2, used in this study were also exploited in a study by Duan et al. [64], albeit that the concentration of photoinitiator was higher in their study.

### 3.3. Fourier Transform Infrared Spectroscopy

The printed dosage forms were characterized in order to evaluate the efficiency of the SLA printing process as the IR spectral changes that occurred during the photopolymerization process could be evaluated. This evaluation is possible as the alterations, peak shifts, which occur in the spectra are indicative that bonding has occurred between the individual constituents present in the photopolymer solution, therefore suggesting that crosslinking has occurred within the samples [57].

The double bond acrylate groups present at the end of the PEG chain in the PEGDA molecule is what gives rise for the polymer to undergo free radical polymerization in the presence of the TPO photoinitiator resulting in the fabrication of the dosage form. The UV light of the SLA printer activates the TPO, resulting in the TPO decomposing to generate two radicals, as previously shown in Figure 2. The free radicals are then available to react with the PEGDA monomer in the photopolymer solution resulting in the opening of the C=C bond present in the PEGDA monomer thus allowing a polymer network to be formed [65]. From conducting FTIR analysis of PEGDA (data not shown) the acrylate groups, C-H bending of the CH_2_=CH–C=O and C–O–C stretching at 811 cm^−1^ and 1189 cm^−1^ respectively were identified with similar findings being reported elsewhere [66,67,68].

**Figure 5:**
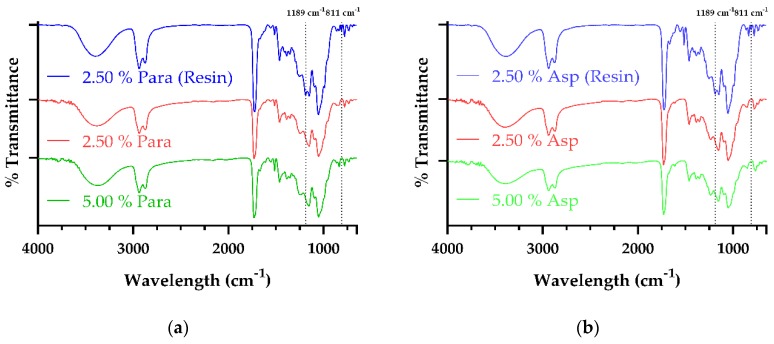
FTIR spectra of (**a**) 2.50% Para (Resin), 2.50% Para dosage form and 5.00% Para dosage form (**b**) 2.50% Asp (Resin), 2.50% Asp dosage form and 5.00% Asp dosage form.

Figure 5a shows the results obtained from FTIR analysis of the paracetamol loaded dosage forms with the spectra for the aspirin samples being displayed in Figure 5b. The FTIR spectra of all printed samples, paracetamol and aspirin, were similar to one another, however, due to the higher ratio of PCL Triol present within the composition, there was over-saturation of the notable peaks of PCL Triol (data not shown) found in printed samples.

As already stated, the higher ratio of PCL Triol within the photopolymer solution resulted in the spectra predominantly appearing as PCL Triol, with the broad peak ranging from 3394.19 to 3369.64 cm^−1^ in the samples being indicative of the free hydroxyl group (–OH) of the PCL Triol while the strong peak ranging from 1729.63 to 1726.05 cm^−1^ is characteristic of the stretching carbonyl (C=O) group of the molecule. It can also be observed that when both the 2.50% Para Resin and 2.50% Asp Resin is compared to their respective printed dosages there is the disappearance of peaks at 811 cm^−1^, and 1189 cm^−1^ with similar findings being reported by Killion et al. [69] and Burke et al. [66,67] reporting that polymerization of a PEGDA derivative, PEGDMA, had occurred upon disappearance of the aforementioned peaks. This finding indicates that the SLA process undertaken within this study may be beneficial within the area of personalized medicine owing to its ability to cure the photopolymer solution and produce dosage forms of not only different active ingredients but also at different drug loading concentrations.

### 3.4. Thermal Characterization

**Figure 6:**
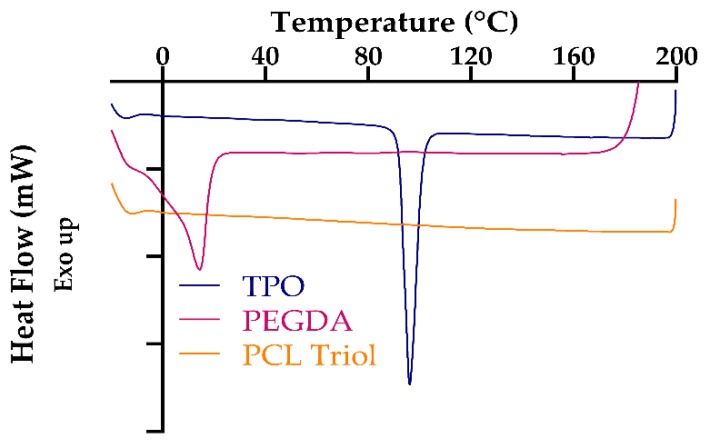
Overlaid DSC thermograms of starting materials.

The study of the thermal properties of the stereolithographic printed tablets and materials used in the fabrication of such tablets was evaluated by the utilization of DSC. Figure 6 shows the DSC thermograms of the materials used in the fabrication of the printed dosage forms. It can be observed that the photoinitiator, TPO, demonstrates a peak a melting peak around 92 °C, which corresponds to the melting point that is found in the materials technical data sheet [70]. The DSC of PEGDA displays an endotherm which is clearly identifiable at 14 °C, which is similar to what Martinez and researchers [22] found, establishing the melting point of PEGDA to be ca. 10 °C from the research that they conducted.

**Figure 7:**
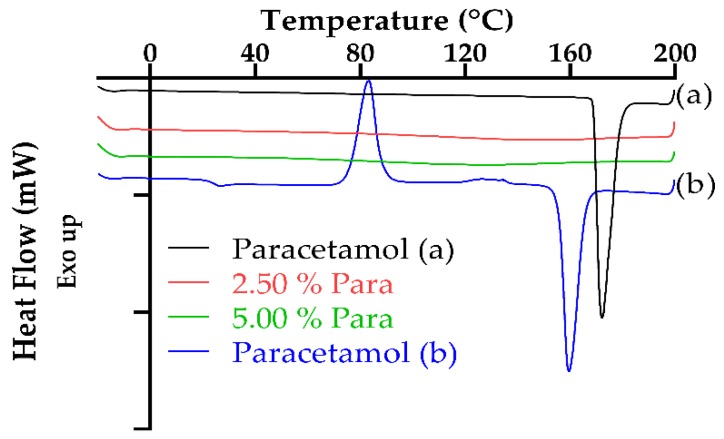
Overlaid DSC thermograms of paracetamol and printed paracetamol dosage forms. (**a**) first heat cycle, (**b**) second heat cycle.

Figure 7 displays the DSC thermograms for paracetamol (first and second heat cycles) and the two printed dosage forms which contain paracetamol. The DSC for paracetamol, (a) first heat cycle, shows a sharp endothermic peak at 169 °C with this melting point being in agreement with that which is found in the literature [5,71]. Furthermore, this melting point is indicative of paracetamol existing in its most stable form, that being the commercially marketed version, Form I (monoclinic) [72,73]. This is further confirmed upon observing the second heat cycle (b) as the melting point has shifted to a lower temperature of 159 °C with Sacchetti [72] noting similar results. Also, when the two heat cycles are compared, there is no cold crystallization peak noted in the first heat cycle, thus indicating that the paracetamol is present in its 100% crystalline form. However, upon observing the second heat cycle, it is apparent that the drug is 100% amorphous (Form II) with a glass transition temperature at 24 °C, with this finding been consistent with that of the literature [73,74]. Furthermore, the broad exothermal peak between 75 and 100 °C, with peak maxima around 80 °C, is typical of cold crystallization occurring with Rengarajan and Beiner [73] reporting results to support this. In addition to this, Rengarajan and Beiner [73] also indicate that the Form II crystals melt at 157 °C with the results of this study finding the onset to be at 155 °C with a maximum melt peak at 159 °C, which according to the literature the melting temperature ranges from 154 to 160 °C which is characteristic of Form II, orthorhombic paracetamol [71].

**Figure 8:**
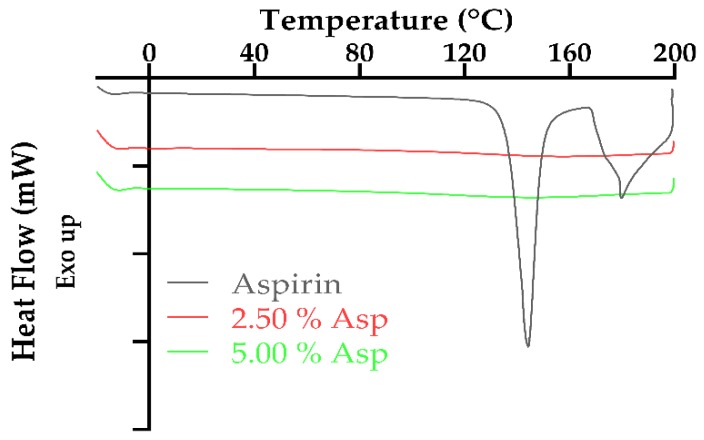
Overlaid DSC thermograms of aspirin and printed aspirin dosage forms.

The overlaid DSC thermograms of aspirin and printed dosage forms containing aspirin can be observed in Figure 8. The DSC for aspirin shows two melt peaks with the first endothermic peak being sharp and present at 144 °C (onset temperature 137 °C) with similar results being reported in the literature [75,76,77]. However, the second endotherm was noted at 179 °C (onset temperature 177 °C) may be attributed to the degradation of aspirin. There are various publications within the literature, [78,79,80], indicating that thermal degradation/decomposition of aspirin occurs in two processes with the first process ranging from 160 to 260 °C. Furthermore, thermogravimetric analysis (TGA) studies conducted by Al-Maydama et al. [81] and de A. Silva et al. [82] deduced that the temperature of maximum degradation (T_DTG_) of aspirin occurred around 181 °C with further DSC studies conducted by de A. Silva et al. [82] confirming this result.

As can be observed in both Figure 7 and Figure 8, there was no endotherm observed for the dosages which contained either drug, paracetamol or aspirin, and either concentration, 2.50% or 5.00%, which is indicative of the active drug being entirely dissolved within the photopolymer solution. Similar results illustrating drug dissolution in the SLA photopolymer solution have been established with a number of publications [20,22,62] citing comparable findings.

### 3.5. Tablet Characterization

**Figure 9:**
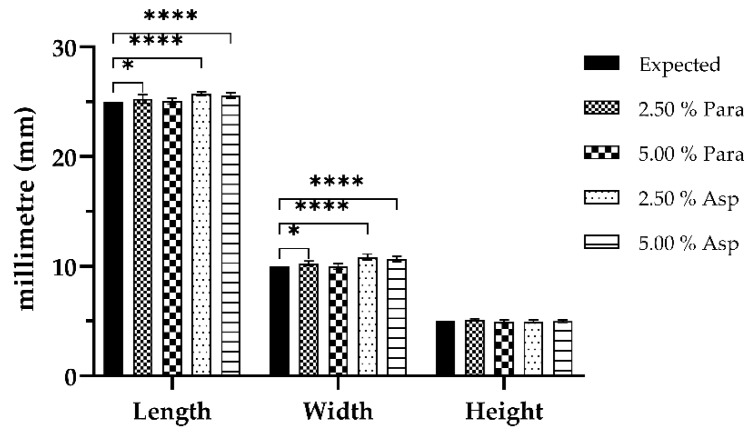
Dimensional analyses values of the paracetamol and aspirin printed dosage forms (*n* = 10, expected values; length 25.00 mm, width 10.00 mm, height 5.00 mm, * = *p* < 0.05, **** = *p* < 0.0001).

In order to evaluate the effectiveness of SLA as a suitable means of fabricating tablets to be utilized in the area of personalized medicine, both the dimensional accuracy of the printing process and uniformity of tablet weight were evaluated. The tablets were fabricated with a high degree of repeatability with respect to their physical dimensions and this was apparent due to the paracetamol and aspirin printed dosage forms producing results close to target dimensions: Length 25.00 mm, width 10.00 mm, and height 5.00 mm, as shown in Figure 9 and Table 2. However, some statistically significant differences were observed when the printed tablets were compared to that of their target dimensions. It was found that the 2.50% Para dosage forms displayed significant differences (*p* < 0.05) in both their length and width when compared to that of their target dimensions. In addition to this, significant differences (*p* < 0.0001) in both length and width were also observed for both the 2.50% Asp and 5.00% Asp dosage forms. Furthermore, there were no significant differences (*p* > 0.05) noted for the 5.00% Para dosage forms in their length and width when compared to that of the target dimension as well as no significant differences (*p* > 0.05) observed for any of the printed dosage forms irrespective of drug or drug loading in regard to the height of the fabricated dosage forms when compared to the expected dimension. The findings presented here are representative of what has previously been outlined in the literature. In two separate studies by Martinez et al. [22,54], the authors assessed the dimensional accuracy of SLA printing as a means of fabricating dosage forms for drug delivery and concluded it was achievable to produce prints with good uniformity in physical dimensions.

**Figure 10:**
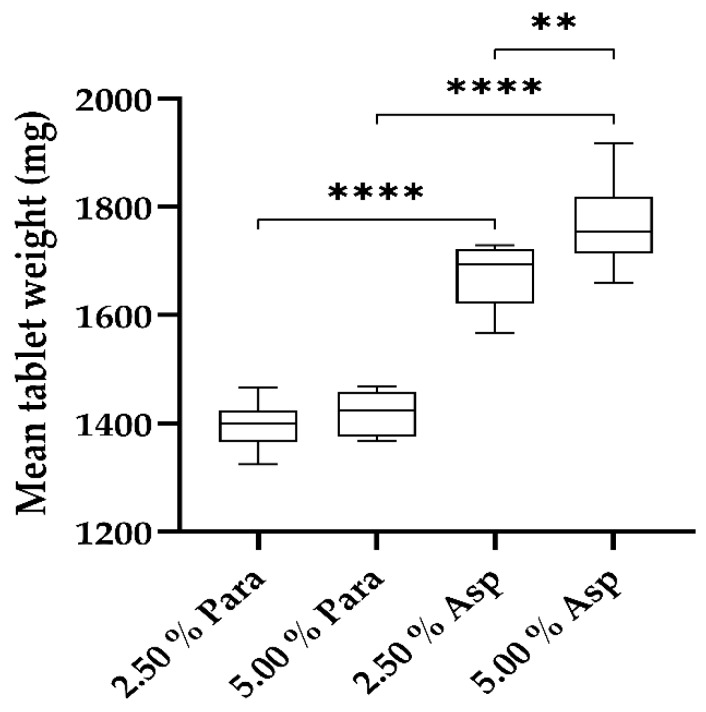
Weight uniformity of paracetamol and aspirin dosage forms (*n* = 10, ** = *p* < 0.002, **** = *p* < 0.0001).

Further to the dimensional analysis being conducted, weight uniformity among the printed tablets was also evaluated with the results presented in Figure 10. Although the absolute weights of the two paracetamol formulations vary slightly from one another, all printed tablet weights ranged from 1324 to 1467 mg with no statistically significant difference (*p* > 0.05) between the two formulations regarding tablet weight observed. However, it was found that the absolute weights of the two aspirin formulations did exhibit some variation, with the printed tablet weights ranging from 1566 to 1918 mg and the differences in tablet weight being significant (*p* < 0.002).

In addition to comparing the printed dosage forms containing the same drugs, the tablets containing the same drug loadings, 2.50% or 5.00%, were also evaluated with respect to uniformity of weight. The printed dosage forms which contained a drug loading of 2.50% showed a significant difference in weight (*p* < 0.0001). Similarly, when the drug loading was 5.00% in the printed tablets they also showed a significant difference (*p* < 0.0001).

Overall, the weight uniformity of all the printed tablets, paracetamol and aspirin, ranges from a lower range of 1324 mg (paracetamol) to an upper range of 1918 mg (aspirin) with significant differences between some tablets being shown as previously outlined with the exception of significant differences between 2.50% Para and 5.00% Para.

These findings reported here of dimensional accuracy and weight uniformity as well as the observations reported in the literature [22,45,54,83,84,85,86] illustrate that AM and in particular the SLA process has the potential to be exploited in the fabrication of dosage forms for a variety of medical conditions. This is evident in the ability to print dosage forms comprised of various monomer(s)/photoinitiator(s)/drug concentrations and/or combinations of each which show excellent consistency in their dimensions with reference to the STL file that was imported into the software of the printer. Furthermore, the results obtained in this study indicate that it was feasible to fabricate a large batch of tablets in a single print, Figure 3a, with 28 dosage forms being produced. In addition to this finding, it should also be noted that a greater number of tablets could be attained through manipulation of the software which would be advantageous when treatment is required more than once per day.

### 3.6. Dissolution Analysis

**Figure 11:**
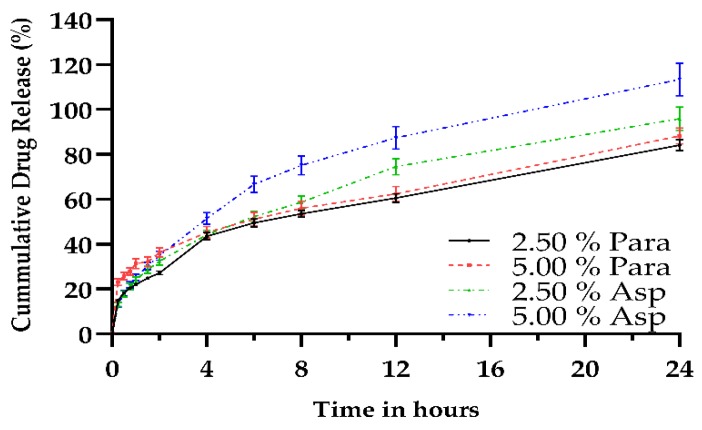
Drug release profiles of both paracetamol and aspirin printed dosage forms over a 24 h period in pH 1.2 (*n* = 6).

Drug release studies were conducted for all of the printed dosage forms with the results shown in Figure 11. As can be observed both the paracetamol and aspirin loaded dosage forms exhibited sustained drug release which was maintained for the duration of the study, 24 h.

Looking at Figure 11, it can be observed that the printed paracetamol dosage forms displayed sustained release of the active drug over the 24 h testing period. It was found that the 2.50% Para exhibited a maximum release of 84.11% with the 5.00% loading achieving a release of 88.16% for the test duration. Over the test period it was found that upon comparison of the two different drug loadings there was statistical significance (*p* < 0.0001) up to the two hour time point after which no statistical significance (*p* > 0.05) was noted until the last and final time point, 24 h, (*p* < 0.002).

As with the paracetamol dosage forms, the aspirin dosage forms also exhibited a sustained rate of release from the 3DP matrices. However, there was a difference in the maximum release after 24 h with the 2.50% Asp having a maximum release rate of 95.85% after the 24 h period with the higher loading, 5.00% Asp, achieving a higher maximum release rate of 113.30% after the same time. However, although the 5.00% Asp dosage form released a greater amount of drug after 24 h, this is believed to have been due to the higher loading of the drug, and therefore less polymer within the polymer matrix. Furthermore, the rate at which aspirin was released from the printed dosage forms was not statistically significant (*p* > 0.05) for time points up to 4 h.

The results presented in this study differ from that reported by Wang and researchers [41] who describe that by altering the ratio of PEGDA to PEG it was possible to modulate drug release, of paracetamol and 4-amino salicylic acid, from the printed dosages with a low percentage of PEGDA (35%) showing faster release rates from that of their higher counterparts. The percentage of PEGDA which was utilized in this study was lower than what was used in the study by Wang et al. [41] with slower release rates been observed with a lower PEGDA content. It was found in the study by Wang and researchers [41] that at the 8 h (480 min) time point that close to 95% of paracetamol was released while at the same point in this study there was only 53% and 56% released from the 2.50% and 5.00% respective paracetamol loaded dosage forms. However, one explanation for this is the incorporation of PEG in the matrix of the dosage form as due to its hydrophilic nature and its inability to crosslink would allow for a reduction in the intermolecular forces between the polymer chains resulting in greater drug release [22]. Additionally, the results also conflict with what was presented by Vehse et al. [87] who incorporated various concentrations of aspirin into PEGDA matrices via a similar technology albeit a different wavelength, and found that more than 95% of aspirin was released within the first 3 h regardless of the drug loading concentration.

The lower release rates established in this study may be attributed to the higher PCL Triol content of the resin composition used in the formulation, with the PCL Triol influencing drug release due to the hydrophobic nature it affords. Asikainen et al. [62] noted that an increase in hydrophobic networks will result in slower drug release. Furthermore, the addition of low molecular weight molecules added to polymer matrices have been reported to have the ability to modulate drug release due to a reduction in the polymer-polymer interactions as a result of the plasticization effect [32,36,37]. Kanis et al. [34] conducted a study evaluating the effect of PCL Triol content imparted on drug release from a poly(ethylene-co-methyl acrylate) matrix and found that by increasing the amount of PCL Triol resulted in a lower amount of drug being released from the matrix. Therefore, it could be possible to control and manipulate the rate at which drug is released from the dosage form by adjusting the ratio of PCL Triol to PEGDA in the formulation, which would be advantageous if immediate release was a requirement of the dosage form.

### 3.7. Scanning Electron Microscopy

**Figure 12:**
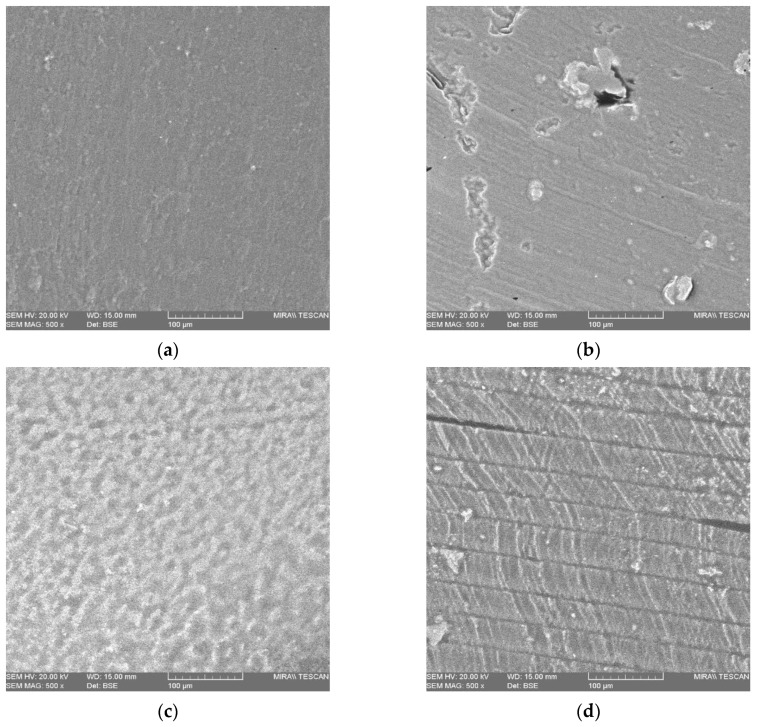
Scanning electron microscopy image of (**a**) 2.50% Para surface (**b**) 2.50% Para cross-section (**c**) 2.50% Asp surface and (**d**) 2.50% Asp cross-section all at 500× magnification.

Scanning electron microscopy (SEM) images of selected paracetamol and aspirin printed dosage forms are presented in Figure 12a–d.

From observing the images on the surface of the paracetamol loaded dosage forms, Figure 12a, it can be seen that there are no visible voids present indicative of the photopolymer resin curing to a high degree which was further confirmed via FTIR analysis, with the disappearance of the peaks at 811 cm^−1^ and 1189 cm^−1^ associated with polymerization occurring [67]. However, in the images displaying the cross-section of the 2.50% Para dosage form, Figure 12b, it appears that there are notable crystals present which may be TPO and/or paracetamol, suggesting that some of the crystals may not have dissolved in their entirety in the photopolymer solution.

The images obtained for the aspirin dosage forms however, Figure 12c,d, appear different to those of the paracetamol dosage forms. The image showing the surface of the 2.50% Asp dosage form, Figure 12c, the surface appears coarse in comparison to that of the paracetamol dosage form which may be as a result of undissolved TPO and/or aspirin settling on the surface of the print with further analysis that is beyond the scope of this work being required to ascertain this finding. In the cross-sectional images of the 2.50% Asp dosage forms, Figure 12d, the layering of the printing process/laser pass can be observed which demonstrates that polymerization of resin occurred with the binding of the resin in a layer-by-layer fashion as would be expected in the SLA process. However, in addition to this, in the 2.50% Asp dosage form there is evidence of voids present, indicating that incomplete curing may have occurred in these areas. Furthermore, there was also particles observed in the 2.50% Asp dosage form which may be present as a result of the TPO and/or aspirin not fully dissolving into the photopolymer solution.

The results found from conducting SEM demonstrate that SLA has the potential to fabricate dosage forms from photocurable resins, with little to no voids being observed on the surface of the dosage forms being indicative of a high level of curing. However, upon closer inspection and viewing the cross-sectional images of the dosage forms, it was found that there may have been incomplete dissolution or agglomeration occurring due to the inhomogeneous distribution of either drug and/or photoinitiator within the resin as crystals were noted. This may be a potential drawback to SLA as an AM technique for drug delivery applications with these findings requiring further investigation in order to elucidate if the observed particles are that of drug and/or photoinitiator.

## 4. Conclusions

The results which are laid out in this paper, like many others in the area of 3DP, illustrate the promising potential the technology offers with respect to dosage form production. The present study harnessed the polymerization of photocurable polymers to fabricate 28 drug dosage forms in one single print cycle, exploiting the additive manufacturing technique of stereolithography for bulk manufacture. The study also highlighted that the incorporation of the different drugs, paracetamol and aspirin, may have had an impact on the overall dimensions of the printed dosage forms, with statistically significant differences observed from their target dimensions. This finding indicates that this is an area which can be further investigated and improved upon in future work to advance the AM technique of SLA in relation to fabrication of personalized dosage forms. In addition to this, significant differences were noted in the mean tablet weight between the paracetamol and aspirin dosage forms, with the aspirin based device having a greater tablet weight which correlated with greater drug release than that of the paracetamol dosage forms. Additionally, as the drug loading was increased, there was also a greater release of active, paracetamol or aspirin, noted indicating that with specific drug loadings it may be possible to formulate patient-specific dosage forms, and that with fine-tuning it may be possible to further modulate drug release. The results presented here demonstrate that 3DP for drug delivery can provide opportunities for bespoke treatment of patients coupled to batch manufacturing. Future work to progress the SLA tablet printing approach must focus on elucidating the differences between traditional tableting, commercially available tablets based on additive manufacturing, and the photocurable polymer formulations described herein. Particular focus is needed on tablet physical properties such as hardness and friability, toxicity related to residual unreacted monomers or solvents, and the stability/shelf-life of photocurable formulations prior to printing.

## Figures and Tables

**Table 1:** Composition of SLA formulations prepared.

SLA Formulation ^1^	PEGDA ^2^	PCL Triol ^3^	Irgacure TPO ^4^	Paracetamol	Aspirin
	% (*w/w*)				
2.50% Para	19.30	77.20	1.00	2.50	-
5.00% Para	18.80	75.20	1.00	5.00	-
2.50% Asp	19.30	77.20	1.00	-	2.50
5.00% Asp	18.80	75.20	1.00	-	5.00

^1^ Stereolithography, ^2^ Poly(ethylene glycol) diacrylate, ^3^ Poly(caprolactone) Triol, ^4^ Diphenyl(2,4,6-trimethylbenzoyl)phosphine oxide.

**Table 2:** Measured parameters of paracetamol and aspirin dosage forms (*n* = 10, expected values; length 25.00 mm, width 10.00 mm, height 5.00 mm).

SLA Formulation	Length (mm)	Width (mm)	Height (mm)
2.50% Para	25.26 ± 0.40	10.27 ± 0.20	5.12 ± 0.10
5.00% Para	25.05 ± 0.26	10.00 ± 0.27	4.96 ± 0.15
2.50% Asp	25.72 ± 0.18	10.85 ± 0.26	4.97 ± 0.11
5.00% Asp	25.58 ± 0.26	10.64 ± 0.25	5.00 ± 0.12
